# A Ratiometric Fluorescent Sensor for the Detection of Norfloxacin in Foods Based on ZIF-8 Core–Shell-Structured Molecularly Imprinted Encoded Microspheres

**DOI:** 10.3390/polym16233351

**Published:** 2024-11-29

**Authors:** Ya Chen, Xueyong Qiao, Guoran Sun, Zhonghui Han, Lei Lv, Xiaolei Zhao, Jinxing He

**Affiliations:** Shandong Key Laboratory of Healthy Food Resources Exploration and Creation, School of Food Science and Engineering, Qilu University of Technology (Shandong Academy of Sciences), Jinan 250353, China

**Keywords:** fluorescent sensor, molecularly imprinted polymer, norfloxacin, metal–organic framework, quantum dots

## Abstract

The development of fluorescent sensors with high sensitivity and fast response times is attracting the interest of more and more researchers. Herein, dual-emission ratiometric molecularly imprinted fluorescent encoded microspheres were fabricated and applied for the fast detection of norfloxacin. Core–shell-structured imprinted polymers with ZIF-8 as the supporting core were obtained first and two quantum dots with green and red emission provided the fluorescent signal. The introduction of the optical encoding technique greatly simplified the preparation process. After the addition of NOR, the green intensity at 525 nm remained constant and the fluorescent intensity at 625 nm decreased significantly because of the inner filter effect. Under the optimum detection conditions, a good linear correlation ranged from 5 μg L^−1^ to 500 μg L^−1^, and the spiked recoveries of the method were 89.76%–106.94%. The detection limit for chicken, pork, fish, and milk samples was established at 2 μg L^−1^. More importantly, the established sensor provided a faster mass transfer rate, and the detection process took only 15 min, indicating great potential as an alternative for the fast detection of NOR in food samples.

## 1. Introduction

Optical sensors have been considered some of the most promising and attractive technologies in the field of food safety detection because of their simplicity of operation, fast response times, and low cost [1,2]. Fluorescent sensors have garnered significant attention owing to their excellent practicality and real-time performance; the fluorescent signals can be evaluated directly without the need for a reference beam, making them more suitable for rapid detection [3].

To improve the accuracy and sensitivity of fluorescent sensors, researchers presented the concept of dual-emission ratiometric fluorescent sensors with simultaneous generation of two distinct emission peaks at an excitation wavelength, which minimized the background interference through self-calibration [4]. Additionally, the molecular imprinting technique is a well-established technology for providing the specific adsorption and high selectivity for templates [5,6]. Combined with molecularly imprinted polymers (MIPs), dual-emission molecularly imprinted fluorescent polymers not only provide the advantages of a fluorescent sensor, but also the specific recognition and excellent selectivity of MIPs, and they have been widely applied in the detection of pesticides, antibiotics, and organic pollutants [7,8,9].

Although significant progress has been made, the preparation process for fluorescent imprinted sensors is often complex. In the method reported here, the oil-soluble fluorescent materials are directly added into the MIP reaction system, inducing a low fluorescent intensity because of the long duration of the reaction and limiting the application of water-soluble fluorescent materials because of the polarity incompatibility with the MIP preparation system, except for the functionalization of fluorescent materials or MIPs [10]. In our previous study, we attempted to incorporate the fluorescent encoding technique into the preparation of fluorescent MIPs with a single color. This strategy greatly simplified the preparation process and enabled universal encoding for fluorescent materials with differing polarities. In this study, we extended this approach to the development of dual-emission fluorescent MIPs.

Effectively reducing the detection time is another significant factor in the practical application of fast detection. Core–shell-structured MIPs emerged as novel assemblies that exhibit significant features for effectively enhancing the mass transfer rate in the adsorption and desorption process—compared with solid MIPs—as the imprinted sites are all located in the external imprinting shell layer [11,12]. Banan et al. utilized magnetic core–shell molecularly imprinted polymers (MMIPs) for the extraction of amiodarone from human plasma, and they conducted a comparative analysis of the extraction efficiencies of bulk polymers and MMIPs. The MMIPs exhibited an adsorption capacity of 42.5 μg mg^−1^, which was 16.35 times higher than that of the bulk polymers (2.60 μg mg^−1^) [13]. The enhancement was attributed to the large specific surface area of the core–shell structure of the MMIPs.

The metal–organic-framework material ZIF-8 has a large specific surface area, strong adsorption, high porosity, and straightforward preparation, and it also exhibits excellent biocompatibility and low toxicity [14]. Its unique structure renders it an ideal core material for ratiometric fluorescent sensors. Using ZIF-8 as a “reaction vessel” for encapsulating one of the fluorescent elements enables the efficient separation of the two quantum dots, thereby preventing interference with their fluorescent intensity and ensuring the accuracy of the detection system in practical applications [15].

Norfloxacin (NOR) is one of the third-generation fluoroquinolone antibiotics and is widely used to treat infections of the urinary tract, intestines, and skin caused by certain bacteria [16]. Residues of NOR in animal-derived food, such as milk products, have been widely reported, and the accumulation of NOR in food may be harmful to human health, causing effects such as fetal malformations, renal failure, and drug resistance [17,18]. Therefore, it is important to develop rapid detection methods for NOR residues in foods.

Based on the above considerations, the aim of this research was to introduce a novel approach for the preparation of dual-emission fluorescent molecularly imprinted sensors and to apply them for rapid, accurate, and sensitive detection of NOR in food samples. The characterization of the encoded microspheres, their fluorescent performance, encoding conditions, detection conditions, and practical applications were all evaluated in detail.

## 2. Materials and Methods

### 2.1. Materials

ZnCdSe/ZnS QDs (G-QDs, λ_em_ = 525 nm, 3 mg mL^−1^; R-QDs, λ_em_ = 625 nm, 3 mg mL^−1^) were obtained from Wuhan Jiayuan Quantum Dot Technology Development Co., Ltd. (Wuhan, China). The preparation process followed a method reported in the literature [19]. Zinc nitrate hexahydrate and acrylamide (AM) were acquired from Sinopharm Chemical Reagent Co., Ltd. (Shanghai, China). Norfloxacin (NOR), ofloxacin (OFX), enrofloxacin (ENR), ciprofloxacin (CIP), ethylene glycol dimethacrylate (EGDMA), divinylbenzene (DVB), and 2-methylimidazole were purchased from Aladdin Chemical Reagent Co., Ltd. (Shanghai, China). DVB and AIBN were purified according to the method reported in [20]. All other reagents were used directly without further purification.

The milk, chicken, pork, and fish samples were purchased from a local supermarket in Jinan and used as real samples.

### 2.2. Instrumentation

The polymers were characterized using a transmission electron microscope (TEM; JEM-1400F, JEOL, Tokyo, Japan), X-ray diffraction (XRD; Rigaku, Tokyo, Japan), and an autosorb nitrogen adsorption apparatus (BELSORP-miniX, miciotracBEL, Osaka, Japan). The UV–visible spectra were recorded using a dual-beam spectrophotometer (TU-1901, Beijing Puritan General Instrument Co., Beijing, China). Fluorescent measurements were performed using a fluorescent spectrophotometer (F2700, Hitachi, Tokyo, Japan).

### 2.3. Preparation of G-QD@ZIF-8 Materials with Green Color

The preparation of ZIF-8 was conducted according to the reported method [21]. Quantities of 0.7344 g of zinc nitrate hexahydrate and 0.8106 g of 2-methylimidazole were individually dissolved in 50 mL of methanol using ultrasonic treatment. Subsequently, the 2-methylimidazole solution was gradually introduced into the zinc nitrate hexahydrate solution. After stirring in the dark for 24 h, the obtained suspension was centrifuged. The precipitate was washed with methanol three times and placed in a vacuum drying oven at 40 °C for 8 h. The resultant material was identified as the metal–organic framework ZIF-8.

To obtain the G-QD@ZIF-8 polymers with a single color, 50 mg of ZIF-8 and 300 μL of G-QDs were added to 1.5 mL of hexane, and the mixture was stirred continuously for 10 min. Following centrifugation at 10,000 rpm for 10 min, the precipitate was washed with enough hexane solution to remove the un-bound G-QDs to give the G-QD@ZIF-8 material.

### 2.4. Preparation of G-QD@ZIF-8@MIP@R-QD Microspheres with Dual-Emission Fluorescent Signals

A quantity of 50 mg of G-QD@ZIF-8 polymers was measured into a 50 mL round-bottomed flask containing 20 mL of acetonitrile, and the mixture was then ultrasonicated for 10 min. Subsequently, 0.1 mmol of NOR as a template was introduced into the G-QD@ZIF-8 dispersion solution, along with 0.4 mmol of AM as a functional monomer, 1 mmol of EGDMA and 1 mmol of DVB as cross-linking agents, and 20 mg of AIBN as an initiator. The mixture was then sonicated for 15 min and purged with nitrogen for 15 min. The reaction mixture was then allowed to proceed at 60 °C for 24 h, resulting in the formation of an imprinted layer on the surface of the G-QD@ZIF-8 matrix. After washing with methanol solution, the template NOR was thoroughly removed and the obtained G-QD@ZIF-8@MIP microspheres with a single color were dried at 60 °C to a constant weight.

**Scheme 1:**
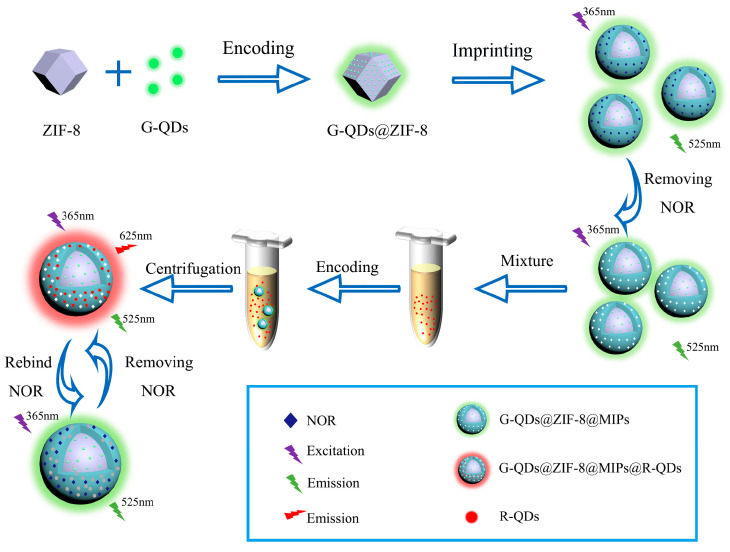
Preparation of dual-emission G-QD@ZIF-8@MIP@R-QD fluorescent encoded microspheres.

Then, a “second” encoding process was carried out. A quantity of 10 mg of G-QD@ZIF-8@MIP microspheres was added to 1 mL of hexane containing 100 μL of R-QD solution. Following ultrasonication for 10 min and stirring for 1 min, the mixed solution was stirred for 1 min, centrifuged at 10,000 rpm for 10 min, and then repeatedly washed with hexane. The final product, denoted G-QD@ZIF-8@MIP@R-QD microspheres with dual-emission signals, was obtained. The preparation process for the dual-emission G-QD@ZIF-8@MIP@R-QD fluorescent encoded microspheres is shown in Scheme 1.

As a control, corresponding non-imprinted polymers (G-QD@ZIF-8@NIPs) were synthesized using the same process, except for the absence of the NOR template. Furthermore, to confirm the advantages of the core–shell structure, traditional core imprinted polymers (S-MIPs) were also prepared without the addition of ZIF-8.

### 2.5. Detection Procedure of the Fluorescent Sensor

The G-QD@ZIF-8@MIP@R-QD microspheres, serving as a fluorescent probe for NOR, were dispersed in acetonitrile at a concentration of 1 mg mL^−1^.

Different concentrations of NOR were added to the above fluorescent probe to achieve the final NOR concentrations of 1, 2, 5, 10, 50, 100, 200, 300, 400, and 500 µg L^−1^. The mixture solutions were shaken for 15 min at room temperature, and the fluorescent spectra were recorded using an excitation wavelength of 365 nm.

The fluorescent intensity was the average of three independent measurements.

### 2.6. Selectivity Experiment

In the control experiments, structural analogues (OFX, ENR, and CIP), amino acids (Phe, Leu, Gly, Cys, and Met), and ions (Al^3+^, Ca^2+^, Fe^2+^, Fe^3+^, K^+^, Na^+^, Zn^2+^, Cl^−^, and NO_3_^−^) commonly found in foods were separately added into the G-QD@ZIF-8@MIP@R-QD and G-QD@ZIF-8@NIP@R-QD fluorescent systems, and the fluorescent intensity changes ((I_625_/I_525_)_0_/(I_625_/I_525_)) were recorded. The mass concentrations of these interfering compounds and NOR were 10 mg L^−1^ and 500 µg L^−1^.

### 2.7. Analysis of NOR in Real Samples

For each blank sample, 1.0 g or 1.0 mL was extracted using 10 mL of acetonitrile with continuous shaking for 10 min and then centrifugated for 10 min at 7000 rpm. The extraction procedure was repeated three times. Then, 10 mL of saturated hexane solution in acetonitrile was added to the collected supernatant solution to remove the fat layer. Then, the obtained acetonitrile layer was evaporated to near dryness and re-dissolved in 1 mL of acetonitrile for fluorescent detection. Meanwhile, spiked recovery experiments were performed by adding NOR standard samples of 0, 5, 10, and 50 μg L^−1^/μg kg^−1^.

## 3. Results and Discussion

### 3.1. Characterization of the Fluorescent Encoded Microspheres

**Figure 1:**
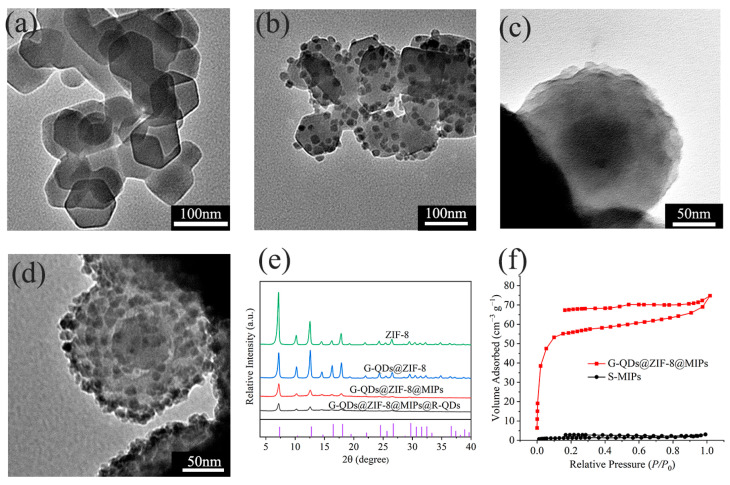
TEM images of (**a**) ZIF-8, (**b**) G-QD@ZIF-8, (**c**) G-QD@ZIF-8@MIP, and (**d**) G-QD@ZIF-8@MIP@R-QD microspheres. (**e**) XRD patterns of obtained polymers. (**f**) Adsorption–desorption isotherms for G-QD@ZIF-8@MIP and S-MIP microspheres.

TEM analysis was conducted to confirm the inner structure of ZIF-8 and the fluorescent encoded microspheres, as shown in Figure 1. In Figure 1a, it can be observed that ZIF-8 exhibited a distinct rhombic dodecahedral structure, which is consistent with previous reports in the literature [22,23]. After mixing with G-QDs, amounts of “black dots” were clearly observed in the inner ZIF-8, and the structure of the G-QD@ZIF-8 polymers maintained the characteristic shape of ZIF-8 (see Figure 1b), confirming the successful loading of the G-QDs. After that, the imprinted layer was prepared on the surface of ZIF-8 and was clearly observed in the TEM image of G-QD@ZIF-8@MIP microspheres, which provided the specific adsorption sites for NOR and served as the encoding matrix for the following, “second” encoding process (see Figure 1c). Furthermore, after secondary encoding of G-QD@ZIF-8@MIP microspheres with R-QDs (λ_em_ = 625 nm) (as shown in Figure 1d), additional “black dots” of R-QDs were observed on the imprinted layer, indicating the successful preparation of dual-emission molecularly imprinted fluorescent encoded microspheres.

XRD analysis was performed on the synthesized polymers, and the results are presented in Figure 1e. The characteristic peaks located at 2θ = 7.3°, 10.3°, 12.7°, 14.6°, 16.4°, and 17.9° corresponded well to the ZIF-8 simulation data from the Cambridge Centre for Structural Data (CCDC 602542) and were also observed for the ZIF-8, G-QD@ZIF-8, G-QD@ZIF-8@MIP, and G-QD@ZIF-8@MIP@R-QD microspheres [24,25]. The results not only indicate the successful preparation of ZIF-8, but also demonstrate the effective introduction of ZIF-8 into the fluorescent microspheres.

Furthermore, nitrogen adsorption–desorption isotherms were used to evaluate the porosity of the G-QD@ZIF-8@MIP and S-MIP microspheres (see Figure 1f). Based on the Brunauer–Emmett–Teller (BET) theory, the specific surface area of G-QD@ZIF-8@MIPs was 203.51 m^2^ g^−1^, which is much higher than that of S-MIPs (4.51 m^2^ g^−1^). The higher specific surface area indicates that G-QD@ZIF-8@MIP microspheres have a greater loading efficiency for QDs and promote the penetration of more QDs deep into the MIP layer, providing encoded microspheres with greater brightness [26].

### 3.2. Evaluation of Fluorescent Properties

**Figure 2:**
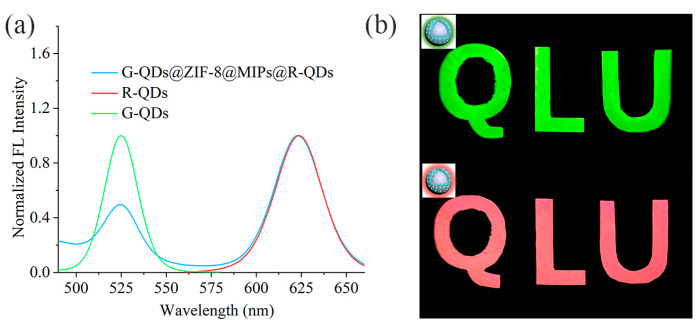
(**a**) Fluorescent spectra of pure G-QDs, R-QDs, and G-QD@ZIF-8@MIP@R-QD microspheres. (**b**) True colors of primary encoding (G-QD@ZIF-8, green) and secondary encoding (G-QD@ZIF-8@MIP@R-QD, pink) under 365 nm UV light.

In this study, G-QDs (λ_em_ = 525 nm) and R-QDs (λ_em_ = 625 nm) were selected as the fluorescent elements of the dual-emission fluorescent system. As shown in Figure 2a, the fabricated G-QD@ZIF-8@MIP@R-QD fluorescent system exhibited well-resolved individual emission peaks. Compared with the original pure G-QDs and R-QDs, the photoluminescence peak position was slightly blue-shifted at 625 nm within 2 nm owing to the accumulation of a few R-QDs.

When exposed to a UV lamp at 365 nm, as shown in Figure 2b, the G-QDs in the fluorescent system of single-color G-QD@ZIF-8@MIPs exhibited a bright green color. Following the second encoding process with R-QDs, the fluorescent color changed from green to almost pink. The successful incorporation of G-QDs and R-QDs within the ZIF-8@MIP matrix was evident, and the core–shell structure effectively prevented the interference of G-QDs and R-QDs.

### 3.3. Optimization of Encoding Conditions

#### 3.3.1. The Effect of R-QD Content on Fluorescent Intensity

**Figure 3:**
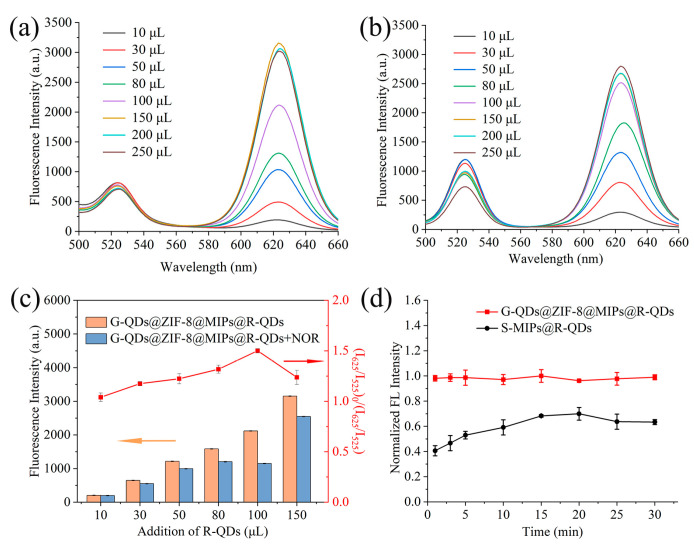
The fluorescent intensity of (**a**) G-QD@ZIF-8@MIP@R-QDs and (**b**) G-QD@ZIF-8@NIP@R-QDs under different R-QD contents. (**c**) The fluorescent intensity and fluorescent response change of encoded microspheres before and after the addition of NOR with different R-QD content incorporations. (**d**) The fluorescent intensity of G-QD@ZIF-8@MIP@R-QD and S-MIP@R-QD microspheres at different times.

The R-QD content was a key factor during the process of fluorescent encoding, mainly influencing the fluorescent intensity and sensitivity of the fluorescent encoded microspheres. At a low QD content, the fluorescent intensity of the encoded microspheres could be limited and not meet the requirement of detection, while too many QDs would induce excessive fluorescent intensity and reduce the sensitivity of the detection method [27]. The relationship between different R-QD contents (10, 30, 50, 80, 100, 150, 200, and 250 µL) and the fluorescent intensity of G-QD@ZIF-8@MIP@R-QD and G-QD@ZIF-8@NIP@R-QD microspheres at 625 nm was investigated. As shown in Figure 3a,b, a progressive enhancement in the fluorescent intensity was observed with the increasing concentration of R-QDs in the range of 10–150 μL, while the fluorescent intensity of G-QDs at 525 nm remained almost constant. This indicated that the G-QDs and R-QDs were located within the ZIF-8 and the molecularly imprinted layer, respectively, which enabled the efficient separation of G-QDs and R-QDs and prevented the effect of direct contact on their fluorescent properties. Meanwhile, it was also found that G-QD@ZIF-8@MIP@R-QD microspheres produced a much greater fluorescent intensity than that of the non-imprinted microspheres when the R-QD content was greater than 100 μL because amounts of R-QDs could be trapped in the imprinted cavities of the imprinted polymers to exhibit stronger fluorescent performance.

To confirm the optimal R-QD content, the fluorescent intensity changes ((I_625_/I_525_)_0_/(I_625_/I_525_)) caused by adding 200 μg L^−1^ NOR to the obtained fluorescent microspheres was also investigated (see Figure 3c). When the amount of R-QDs was in the range of 10–100 μL, the quenching effect gradually increased with increasing amounts of R-QDs, achieving a maximum value at 100 μL, and a decreased fluorescent quenching effect was observed. Therefore, the optimal R-QD content for the subsequent experiments was determined to be 100 μL. At the optimum QD content, the G-QD/R-QD ratio was 3:1.

#### 3.3.2. The Effect of Encoding Time on Fluorescent Intensity

To shorten the encoding time and verify the fluorescent encoding efficiency, we recorded the fluorescent intensity of G-QD@ZIF-8@MIP@R-QDs and S-MIP@R-QDs at different times (1, 3, 5, 10, 15, 20, 25, and 30 min). From Figure 3d, the fluorescent intensity of G-QDs for G-QD@ZIF-8@MIP@R-QD microspheres was 98.15% of its maximum value at 1 min and remained almost at equilibrium. As a comparison, the S-MIP@R-QDs required almost 20 min to achieve encoding. More importantly, the G-QD@ZIF-8@MIP@R-QDs achieved a much higher fluorescent intensity, 1.34 times that of the S-MIP@R-QD microspheres. This result gives direct evidence that the fluorescent system composed of G-QD@ZIF-8@MIP@R-QDs had a faster mass transfer rate and a higher loading capacity of QDs owing to the existence of the core–shell structure of the encoding matrix of the G-QD@ZIF-8@MIPs, which was also shown in the BET analysis [28].

### 3.4. Establishment of the Dual-Emission Fluorescent Sensor

#### 3.4.1. Stability Evaluation

**Figure 4:**
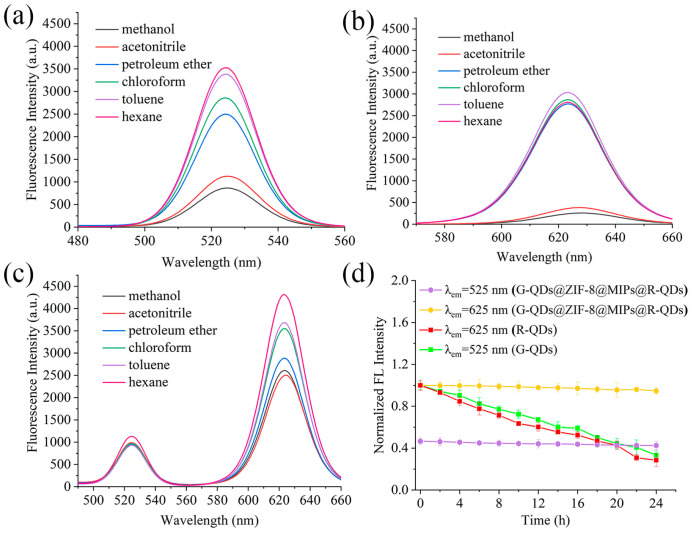
The fluorescent intensity of (**a**) G-QDs, (**b**) R-QDs, and (**c**) G-QD@ZIF-8@MIP@R-QD microspheres in different solvents. (**d**) The fluorescent intensity of G-QD@ZIF-8@MIP@R-QD microspheres and QDs at wavelengths of 525 nm and 625 nm under continuous UV light irradiation.

Because of surface defects and interparticle aggregation, the fluorescent properties of nanosized QDs are unstable and easily influenced by external environmental conditions, such as polar solvents and light exposure [29]. The solvent stability and photo-stability of the G-QD@ZIF-8@MIP@R-QD microspheres were examined in detail and compared with those of pure G-QDs and R-QDs. The results can be summarized as follows:

(1) During the solvent stability evaluation, the fluorescent spectra of the G-QD@ZIF-8@MIP@R-QD microspheres were recorded in various solvents with different polarities (methanol, acetonitrile, petroleum ether, chloroform, toluene, and hexane). As can be seen in Figure 4a–c, the pure G-QDs and R-QDs all exhibited a significant decrease in fluorescent intensity and exhibited a red-shift in their emission wavelengths, particularly in the high-polarity solvents (methanol and acetonitrile). However, the G-QD@ZIF-8@MIP@R-QDs exhibited an almost constant fluorescent intensity at 525 nm and a slight decrease at 625 nm with the increase in solution polarity.
(2) The fluorescent intensity of the abovementioned encoded materials was measured under continuous UV light irradiation for 24 h. As shown in Figure 4d, the remaining fluorescent responses of the R-QDs and G-QDs in the G-QD@ZIF-8@MIP@R-QDs were above 94.66% and 96.01% of the original fluorescent intensity, while those of pure G-QDs and R-QDs decreased to as low as 33.22% and 28.47%, indicating that the G-QD@ZIF-8@MIP@R-QD microspheres exhibited much higher photo-stability than the pure G-QDs and R-QDs.

The above stability evaluation suggests that the imprinted layer provided strong protection for pure G-QDs and R-QDs, reducing the influence of the environmental conditions. The higher stability could ensure the accuracy of the established fluorescent sensor in practical applications.

#### 3.4.2. Fluorescent Quenching Mechanism

**Figure 5:**
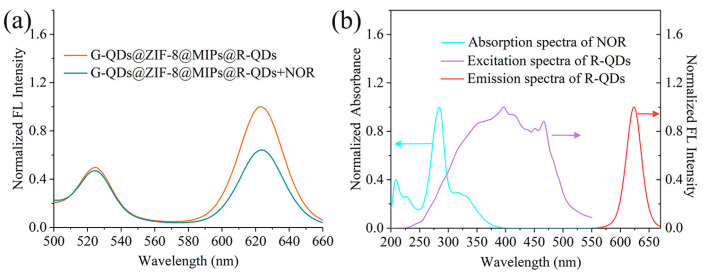
(**a**) Fluorescent spectra of G-QD@ZIF-8@MIP@R-QD microspheres before and after the addition of NOR. (**b**) UV–visible absorption spectra of NOR and fluorescent excitation and emission spectra of R-QDs.

Figure 5a shows the interesting observation that the fluorescent intensity of the 625 nm emission peak of R-QDs decreased and that the green intensity of G-QDs remained almost constant upon the addition of NOR. An investigation was conducted to evaluate the quenching mechanism. As the recorded UV absorption of NOR shows (Figure 5b), NOR exhibits a wide adsorption band at the location of 250–380 nm. When this overlapped with the excitation or emission wavelength of the G-QDs, the fluorescent intensity could be quenched because of the fluorescent resonance energy transfer (FRET) or inner filter effect (IFE) [30]. However, the FRET effect was excluded given the absence of overlap between the UV absorption of NOR and the fluorescent emission spectra of the G-QDs, yet there was a significant overlap of the excitation spectra of the R-QDs and the UV absorption spectra of NOR attributed to the effective IFE [31]. That is, the NOR molecules acted as an energy acceptor to absorb the excitation response of the R-QDs as an energy donor. Based on the above observation, the G-QD@ZIF-8@MIP@R-QD fluorescent system could be applied as a ratiometric fluorescent sensor for quantitative detection of NOR.

#### 3.4.3. Optimization of Detection Conditions

**Figure 6:**
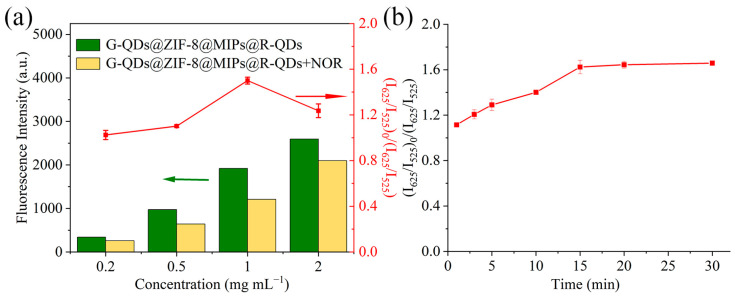
(**a**) Effect of encoded microsphere addition on the fluorescent detection system. (**b**) Time response of G-QD@ZIF-8@MIP@R-QDs for NOR detection.

The detection performance was significantly influenced by the concentration of the encoded microspheres. When the concentration was too low, the established fluorescent sensor had a low fluorescent intensity and a reduced specific adsorption. Conversely, the fluorescent quenching effect could be induced by more analytes at excessive concentrations, resulting in the low sensitivity of the fluorescent sensor. The effect of 200 μg L^−1^ of NOR on the fluorescent intensity of G-QD@ZIF-8@MIP@R-QDs at various concentrations (0.2, 0.5, 1, and 2 mg mL^−1^) was studied. Figure 6a exhibits the maximum fluorescent response change achieved when the microsphere concentration was 1.0 mg mL^−1^. Consequently, 1.0 mg mL^−1^ was chosen as the optimal microsphere concentration and utilized in the subsequent detection process.

At a constant NOR concentration of 200 μg L^−1^, the relationship between the fluorescent response change ((I_625_/I_525_)_0_/(I_625_/I_525_)) of the G-QD@ZIF-8@MIP@R-QD system at different detection times ranging from 1 to 30 min was evaluated, as shown in Figure 6b. As the detection time increased, it can be clearly observed that the fluorescent response change gradually increased and reached equilibrium within 15 min. However, in our previous study, the detection time using solid imprinted fluorescent encoded microspheres as the encoding matrix was almost 30 min and two times that of the G-QD@ZIF-8@MIP@R-QD fluorescent system [27].

The BET analysis, encoding time, and detection time results all demonstrated the advantages of the core–shell-structured imprinted polymers in terms of increasing the adsorption capacity and mass transfer rate. In addition, the G-QD@ZIF-8@MIP@R-QD fluorescent sensor requires a much shorter analysis time, making it more beneficial for rapid detection.

### 3.5. Analytical Performance of the Dual-Emission Ratiometric Fluorescent System

**Figure 7:**
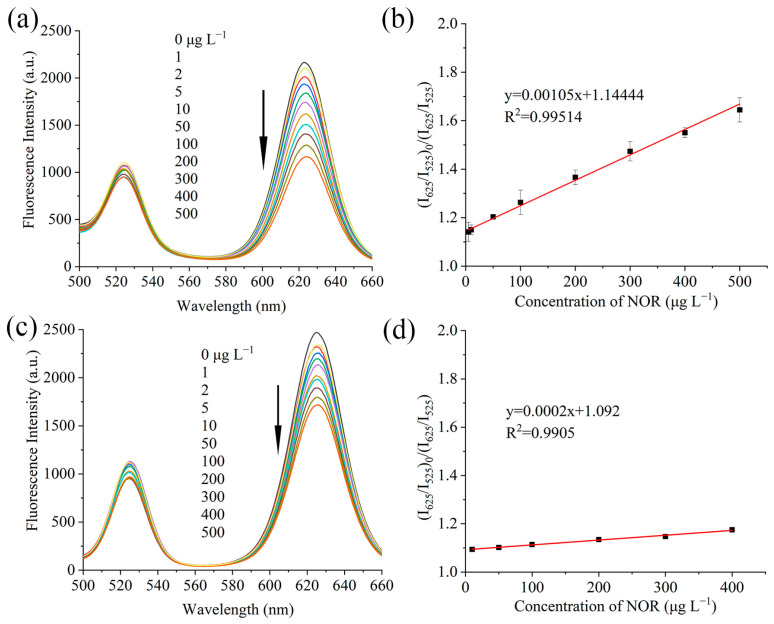
Fluorescent intensity of (**a**) G-QD@ZIF-8@MIP@R-QD and (**c**) G-QD@ZIF-8@NIP@R-QD encoded microspheres after the addition of different concentrations of NOR (0–500 μg L^−1^). Corresponding linear curves for (**b**) G-QD@ZIF-8@MIP@R-QD and (**d**) G-QD@ZIF-8@NIP@R-QD sensors.

With the optimization of detection conditions, a ratiometric fluorescent sensor with G-QD@ZIF-8@MIP@R-QD microspheres as the fluorescent matrix was established. Upon the addition of NOR concentrations ranging from 0 to 500 μg L^−1^, the fluorescent response changes of G-QD@ZIF-8@MIP@R-QDs and G-QD@ZIF-8@NIP@R-QDs were recorded, as shown in Figure 7a,c. When the NOR concentration was increased, the fluorescent intensity at 525 nm was almost constant, while the red intensity at 625 nm underwent a gradual decrease. Based on previous reports, the relationship between fluorescent intensity and concentration could be analyzed using the following Stern–Volmer equation [32]:

$$F_{0}\;/F=K_{\mathrm{SV}}\;C_{\mathrm{NOR}}+1$$

where *F*_0_ and *F* denote the fluorescent intensity of the sensor in the absence ((I_625_/I_525_)_0_) and presence (I_625_/I_525_) of NOR, respectively; *K*_SV_ represents the response coefficient of the equation; and *C*_NOR_ stands for the concentration of NOR.

As shown in Figure 7b,d, the fluorescent response changes of (I_625_/I_525_)_0/_(I_625_/I_525_) for the G-QD@ZIF-8@MIP@R-QD sensor increased linearly with increasing NOR concentrations in the range of 5–500 μg L^−1^, with a coefficient of 0.9954. Notably, because of the existence of imprinted sites, the fluorescent response changes of the G-QD@ZIF-8@MIP@R-QD sensor were much higher than those of the non-imprinted sensor at all measured concentrations, with an excellent imprinted factor of 5.25. The lowest limit of detection (LOD) of the sensor was measured to be 2 μg L^−1^ when the concentration of NOR was reduced to the point where there was no change in the fluorescent signal of the sensor.

### 3.6. Selective Evaluation

**Figure 8:**
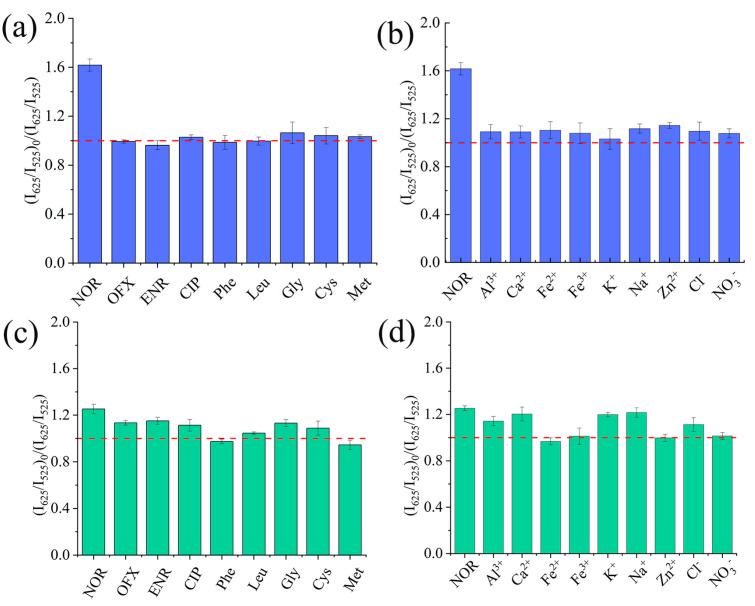
Fluorescent response of G-QD@ZIF-8@MIP@R-QD sensor to (**a**) NOR and its structural analogs and amino acids and (**b**) common ions. Corresponding fluorescent responses of G-QD@ZIF-8@NIP@R-QD sensor to interfering compounds shown in (**c**,**d**).

A selective evaluation of G-QD@ZIF-8@MIP@R-QD and G-QD@ZIF-8@NIP@R-QD fluorescent sensors was conducted, and the results are shown in Figure 8.

As shown in Figure 8a, only NOR showed the highest fluorescent intensity change ((I_625_/I_525_)_0_/(I_625_/I_525_) = 1.617), while OFX, ENR, and CIP only produced a slight fluorescent change, with (I_625_/I_525_)_0_/(I_625_/I_525_) values ranging from 0.96 to 1.02. A similar result was also obtained for the evaluation of the effect of various amino acids (Phe, Leu, Gly, Cys, and Met) and ions (Al^3+^, Ca^2+^, Fe^2+^, Fe^3+^, K^+^, Na^+^, Zn^2+^, Cl^−^, and NO_3_^−^) on fluorescent response change, with an (I_625_/I_525_)_0_/(I_625_/I_525_) value of 0.98–1.14 (see Figure 8b). Meanwhile, we clearly observed that the G-QD@ZIF-8@NIP@R-QD sensor only provided a negligible fluorescent intensity change for the measured NOR and other interfering compounds, with (I_625_/I_525_)_0_/(I_625_/I_525_) values in the range of 0.96 to 1.25 (see Figure 8c,d).

The selectivity experiments confirmed the high selectivity of the G-QD@ZIF-8@MIP@R-QD sensor for NOR, indicating that the imprinted sites were present and fitted exactly with the NOR, while other interfering compounds and the non-imprinted sensor just depended on the slight non-specific adsorption.

### 3.7. Detection of Real Samples

**Figure 9:**
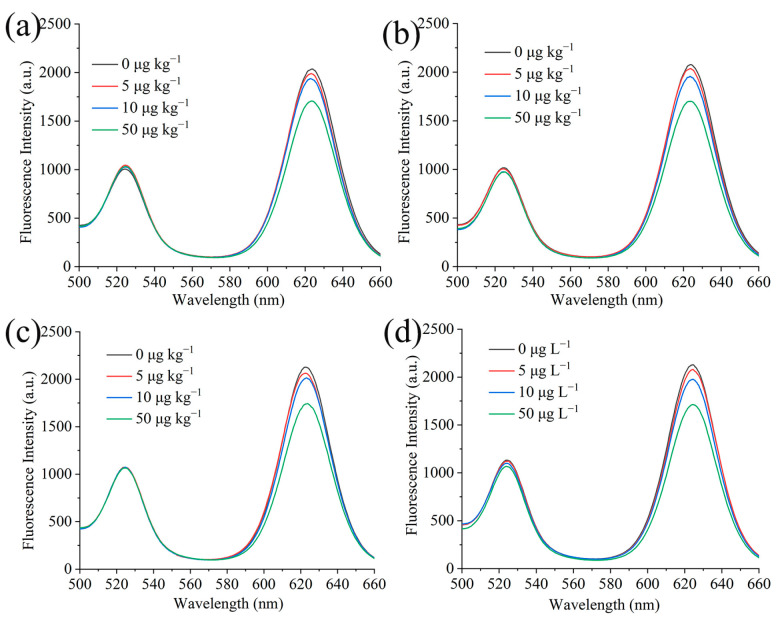
Fluorescent spectra of G-QD@ZIF-8@MIP@R-QD sensor for (**a**) chicken, (**b**) pork, (**c**) fish, and (**d**) milk samples with spiked concentrations of 0, 5, 10, and 50 μg kg^−1^/μg L^−1^.

To verify the feasibility of the established G-QD@ZIF-8@MIP@R-QD sensor for the detection of NOR, spiked recovery experiments were conducted to evaluate its accuracy, using milk, chicken, pork, and fish as real samples. The results showed that the developed sensor had excellent intraday (within 1 day) and interday (within 3 days) precision, with good feasibility in detection. Good recoveries ranging from 89.76% to 106.94% with relative standard deviations (RSDs) of 1.58%–8.96% were obtained (see Table 1), which meet the requirement of quantification analysis. The fluorescent spectra of the real samples are shown in Figure 9. Compared with the currently reported methods listed in Table 2, the fluorescent sensor developed in this study has a wider linear range, shorter detection time, and lower LOD, and it can be applied to a wider range of samples. The results confirmed that the ratiometric fluorescent sensor was successfully applied and is trustworthy for NOR detection in real samples.

## 4. Conclusions

In summary, a dual-emission ratiometric fluorescent sensor was successfully constructed and applied for the detection of NOR in food samples. There are three overwhelming advantages which should be emphasized: (1) The employment of the core–shell structure not only enhanced the mass transfer rate and the loading capacity of the QDs, but provided the effective spatial separation of G-QDs and R-QDs, preventing the direct contact of G-QDs and R-QDs from impacting the fluorescent performance. (2) Due to the fluorescent encoding technique, the preparation process for the dual-emission molecularly imprinted fluorescent encoded microspheres was greatly simplified, which solved the polarity limitation of QDs and solvents and expanded the application of QDs. (3) The constructed ratiometric fluorescent sensor exhibited high accuracy and sensitivity. More importantly, the detection time was significantly reduced to just 15 min, making it more suitable for fast detection. We believe our established method not only provides an effective alternative for fast detection, but, as has been highlighted, also has significant potential in food safety.

## Figures and Tables

**Table 1:** The detection of NOR in different samples.

Samples	Added (μg kg^−1^/μg L^−1^)	Intraday			Interday		
		Found (μg kg^−1^/μg L^−1^)	Recovery (%)	RSD (%)	Found (μg kg^−1^/μg L^−1^)	Recovery (%)	RSD (%)
Chicken	0.00	ND ^a^	-	-	ND ^a^	-	-
	5.00	4.71	94.21	4.03	4.62	92.46	6.31
	10.00	10.19	101.94	2.26	10.32	103.21	3.68
	50.00	49.22	98.44	4.37	48.83	97.66	3.44
Pork	0.00	ND ^a^	-	-	ND ^a^	-	-
	5.00	5.35	106.94	7.87	5.22	104.42	4.99
	10.00	9.52	95.23	1.97	9.55	95.47	3.07
	50.00	46.92	93.84	2.60	44.88	89.76	3.48
Fish	0.00	ND ^a^	-	-	ND ^a^	-	-
	5.00	5.05	101.04	8.86	5.14	102.82	8.96
	10.00	9.78	97.83	4.31	9.35	93.47	4.89
	50.00	46.26	92.52	5.57	47.88	95.76	2.03
Milk	0.00	ND ^a^	-	-	ND ^a^	-	-
	5.00	4.67	93.46	7.54	4.82	96.40	2.88
	10.00	9.48	94.76	8.32	9.52	95.23	6.94
	50.00	47.61	95.21	1.58	49.22	98.44	7.39

^a^ Not detectable.

**Table 2:** Comparison of the proposed sensor with other reported NOR detection methods.

Methods	Samples	Linear Range	Detection Time (min)	LOD	Reference
HPLC	Urine, serum	20.00–120.00 μg L^−1^	-	6.00 μg L^−1^	[33]
Fluorescent probes	Blood	31.80–6880.00 μM	150	10.00 μM	[34]
Nanozymes	NOR capsules	1.25–8.00 μM	30	0.20 μM	[35]
Nanozyme	Water	0.13–2.51 μM 17.5–100 μM	25	0.08 μM	[36]
Fluorescent aptasensor	Milk	50–6000 nM	40	31 nM	[37]
Ratiometric fluorescent sensor	Chicken, pork, fish, milk	5.00–500.00 μg L^−1^	15	2.00 μg L^−1^	This work

## Data Availability

Data are contained within the article.
